# Evaluation of reagents for suspension-assisted total reflection X-ray fluorescence element analysis in biomedical samples

**DOI:** 10.1007/s00216-026-06505-2

**Published:** 2026-04-30

**Authors:** Sebastian Hauser, Marit Veit, Kerstin Leopold

**Affiliations:** Institute of Analytical and Bioanalytical Chemistry (IABC), Ulm University, Albert-Einstein-Allee 11, 89081 Ulm, Germany

**Keywords:** Total reflection X-ray fluorescence spectroscopy, Analytical greenness evaluation, Suspension-assisted sample preparation, Element analysis, Biomedical research, Biological tissue

## Abstract

In biomedical research, it is essential to be able to precisely and reliably quantify trace and microelements in complex biological matrices in order to identify deficiencies and understand disease mechanisms. Analytical methods that are sensitive, robust, and environmentally friendly are preferable. Suspension-assisted total reflection X-ray fluorescence spectrometry (SA-TXRF) is a simple to perform, multielement analytical method that can quantify trace elements in suspensions containing minute sample amounts. In this work, a systematic study comparing five different suspension reagents, namely concentrated and diluted nitric acid, Triton X-100, Triton X-114, and ultrapure water, for SA-TXRF analysis of trace and microelements in three biological reference materials (NIST 1577c, NIST 1486, and ERM-BB186) is presented. The analytical performance is compared in terms of trueness, precision, and sensitivity, and the resulting SA-TXRF methods were assessed for greenness using the Analytical Greenness Metric for Sample Preparation (AGREEprep). As a result, water is recommended as suspension reagent for soft biological material, while for hard biological material, i.e., bone meal, best results were achieved using the surfactant Triton X-114. All of the presented SA-TXRF methods demonstrate improved greenness compared to the standard method (EPA Method 3052), which involves sample preparation by microwave-assisted digestion.

**Graphical abstract:**

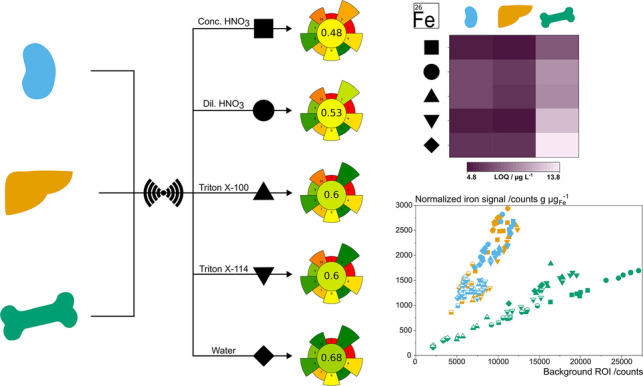

**Supplementary Information:**

The online version contains supplementary material available at 10.1007/s00216-026-06505-2.

## Introduction

The quantitative determination of trace and microelements in biological samples is crucial in biomedical research, as it supports the detection of deficiencies, the elucidation of disease mechanisms, clinical diagnosis and therapy, and the evaluation of exposure, accumulation, and toxicity of pollutants [1–3]. For example, accurate measurement of iron concentrations is essential in studies on the hormonal regulation of iron homeostasis and disorders of iron metabolism [4, 5]. Large-scale determination of trace elements such as zinc, iron, and copper in blood or tissue can also provide valuable insights into population health [6, 7]. Since the concentrations of trace and microelements typically range from about 500 µg g^−1^ down to well below 1 µg g^−1^ [8, 9], analytical methods should ideally offer a wide linear dynamic range and enable simultaneous multi-element detection. Various elemental analytical techniques, including inductively coupled plasma-optical emission spectrometry (ICP-OES), inductively coupled plasma-mass spectrometry (ICP-MS), and total reflection X-ray fluorescence (TXRF), are suitable for such determinations [10–12].

For the analysis of human biological material, only limited sample quantities are often available. The same is true for samples from biomedical research involving animal experiments, which are increasingly viewed critically and should follow the guiding principle of minimizing the number of animals while maximizing the information obtained from each individual specimen. Consequently, non-destructive microanalytical methods are preferred, and where this is not feasible, sample consumption should be kept as low as possible. Another aspect of growing importance in the selection of suitable analytical methods, particularly for monitoring and routine analysis, is their sustainability [13]. Providing green analytical methods is one of the social responsibilities of analysts that aligns with the Sustainable Development Goals (SDGs) of the United Nations 2030 Agenda [14]. The 12 principles of Green Analytical Chemistry (GAC) provide a framework for assessment of greenness of analytical methods [15]. However, the first principle states that direct analytical methods should be favored over multistep sample pretreatment analysis procedures. In the case of trace analysis, direct analysis of the sample without any sample preparation is frequently not possible. Therefore, in these cases, it is more appropriate to consult the 10 principles of Green Sample Preparation for assessment of method sustainability [16]. These principles include, among others, the use of safe, non-toxic solvents and reagents, as well as the minimization of waste and of the amounts of sample, chemicals, and materials required.

An analytical method that can meet the above-mentioned requirements is total reflection X-ray fluorescence spectrometry (TXRF). This technique offers simple trace element quantification by internal standardization and multielement analysis in minute sample amount. Thereby, a few microliters of a homogenate are deposited and dried on a reflective sample carrier. The established sample preparation for TXRF analysis of biological tissues is sample digestion [17–21]. Here, tissues are typically digested using mineral acids and/or acid mixtures at increased temperature. This often involves labor-intensive processes that are also time- and reagent-consuming, see, e.g.,[17] or EPA method 3052 [22]. In order to improve greenness, suspension-assisted total reflection X-ray fluorescence analysis (SA-TXRF) has been introduced in recent years [23, 24]. This method involves finely grinding the sample and suspending it in a suitable solvent, optionally with the addition of a surfactant and/or ultrasonic treatment. Quantification in TXRF can be achieved by the simple addition of an aqueous internal standard to the homogenate enabling convenient one-point calibration. In the case of SA-TXRF, the most critical prerequisites for obtaining accurate analytical results are the particle size of the solid material and the homogeneous distribution of both sample and internal standard on the reflective sample carrier. Furthermore, appropriate transfer of the suspended sample and the influence of the deposition morphology resulting from drying prior to analysis are crucial factors. In addition to ensure analytical accuracy, these methodological aspects are also decisive for reaching lowest possible limits of detection (LODs). Few applications of SA-TXRF to biomedical samples have been reported in recent years, including the determination of iron in human placenta [25], trace elements in mice cells [26], human tissue [27], animal tissues [28], and medieval bones [29]. These studies show that the diversity of biomedical samples necessitates different optimal SA-TXRF approaches. These differences arise not only from varying degrees of sample homogenization in the suspensions, but also from differences in the chemical composition and physical properties of the sample matrix. While biological samples exhibit a great diversity in terms of physical characteristics, matrix composition and complexity and are miscellaneous as the world itself, possible simplifications and classifications can be made. One is the allocation into hard and soft biological matter. Soft biological matter comprises materials with low stiffness that can be severely deformed by forces (e.g., soft tissues) [30], while hard biological matter refers to highly mineralized, mechanically stiff tissues such as bone or dental tissue [31]. Soft biological materials relevant to biomedical research primarily include tissues and blood samples whose main components, aside from water, are organic compounds. In X-ray fluorescence, these are referred to as “dark matrix,” i.e., elements with low atomic numbers that exhibit no or only very low X-ray fluorescence yield. Hard biological materials, by contrast, mainly comprise teeth and bones, whose principal constituent, hydroxyapatite, consists primarily of calcium and phosphorus. When analyzing hard biological materials by SA-TXRF, both the increased hardness during milling and potential line overlaps in the X-ray fluorescence spectrum, mainly arising from calcium as the dominant matrix element, must therefore be taken into account.

The objective of this study is to methodically examine the impact of different suspension media on the SA-TXRF analysis of micro- and trace elements in biomedical samples, with respect to the analytical performance in terms of trueness, precision, and sensitivity. Therefore, three different reference materials are investigated, namely, bovine liver (SRM 1577c), pig kidney (ERM186) and bone meal (SRM NIST 1486), with certified trace element concentrations. To the best of our knowledge, while SA-TXRF has been applied to several soft biological matrices [25–28], only one prior study has addressed trace element analysis in bone using SA-TXRF [29]. Therefore, the present study will emphasize the comparison of hard and soft matrices. This will shed light on matrix-dependent suspension behaviour and help to establish selection criteria for optimising suspension media in future studies. The present study therefore closes a methodological gap by extending suspension-assisted strategies to hard biological matter and validating their analytical performance against established preparation methods. Furthermore, the resulting optimized SA methods are compared to the established digestion-based TXRF method in terms of sustainability using the AGREEprep metric [16].

## Experimental

### Certified reference materials

Three biological reference materials with certified trace element mass fractions were investigated: SRM NIST 1577c (bovine liver; National Institute of Standards & Technology, Gaithersburg, MD, USA), SRM NIST 1486 (bone meal, National Institute of Standards & Technology, Gaithersburg, MD, USA) and ERM-BB186 (pig kidney, Institute for Reference Materials and Measurements, Geel, Belgium). These certified reference materials (CRMs) were chosen because they resemble the types of real-world samples commonly investigated in biomedical research. They represent both soft and hard biological matter, and the concentrations of their trace elements have been certified (s. Table 1). All reference materials were stored and prepared according to the certificate of analysis. NIST 1577c was dried to mass constant in a desiccator over CaSO_4_ (Thermo Fisher Scientific Inc, Waltham, MA, USA) and ERM-BB186 was dried to mass constant in a drying oven (Heratherm oven, Thermo Fisher Scientific Inc, Waltham, MA, USA) at 103 °C. For NIST 1486 a sub-sample was taken simultaneously to sampling to determine a moisture correction factor. Samples were dried at 105 °C for 2 h in a drying oven and the mass loss was determined to be approx. 0.7%.

**Table 1:** Certified reference mass fractions of elements of interest and evaluated in this study in investigated reference materials NIST 1577c, NIST 1486, and ERM-BB186

Mass fraction (mg kg^−1^)	NIST 1577c bovine liver	ERM-BB186 pig kidney	NIST 1486 bone meal
Ca	131 ± 10		
Cu	275.2 ± 4.6	36.5 ± 1.8	
Fe	197.94 ± 0.65	255 ± 13	99 ± 8
K			412 ± 4
Mn	10.46 ± 0.47	7.26 ± 0.25	
Se	2.031 ± 0.045	10.3 ± 0.9	
Zn	181.1 ± 1.0	134 ± 5	147 ± 16
Pb			1.335 ± 0.014

### Sample preparation

Five different suspension reagents were investigated: concentrated nitric acid (65 wt.-%, Normapur, VWR International GmbH; Darmstadt, Germany), diluted nitric acid (12 wt.-%), Triton X-100 (1 vol.-%), Triton X-114 (1 vol.-%), and ultrapure water (UPW; 18.2 MΩ cm, 100 μS cm^−1^, arium pro, Sartorius AG, Göttingen, Germany). Subboiled nitric acid was used as a reagent and in all steps (DST-1000, Savillex Corporation, Eden Prairie, MN, USA). For diluted nitric acid, 1.414 mL concentrated nitric acid was diluted to 10 mL with UPW. For Triton dilutions, 100 µL Triton X-100 (Merck KGaA, Darmstadt, Germany) or Triton X-114 (AppliChem GmbH, Darmstadt, Germany) were diluted to 10 mL with UPW. For preparation of sample suspensions, approx. 10 mg or 20 mg of reference material was weighed into precleaned 2 mL tubes and subsequently 1000 µL of suspension reagent and internal standard solution (100 mg L^−1^) were added. Duplicates were produced of each suspension. For SRM 1577c and ERM-BB146, 25 µL titanium internal standard per 10 mg of sample was added, and for SRM 1486, 10 µL chromium internal standard was added to roughly match mass fractions of elements of interest. Suspensions were vortexed (Digital mini vortex mixer, VWR International GmbH; Darmstadt, Germany) for 60 s at 2500 rpm and placed in an ultrasonic bath (USC 300 THD, VWR International GmbH; Darmstadt, Germany) at 40 °C for 5 min. This procedure was performed once (bone meal) or three times (bovine liver and pig kidney) in total. Shortly before applying an aliquot (10 µL) of the suspension onto a hydrophobized TXRF sample carrier (30 × 3 mm, Bruker Nano GmbH, Berlin, Germany and B&M Optik GmbH, Limburg, Germany), suspensions were vortexed again for 30 s at 2500 rpm. Solvent was evaporated on a heating plate (VMS-C7, VWR International GmbH; Darmstadt, Germany) at 60 °C for 90 min. From each suspension, triplicates of sample carriers were produced. All steps were carried out under laminar flow box class 100 (SuSi Super Silent, Spectec GmbH, Erding, Germany). A scheme summarizing the sample preparation procedure can be found in Figure S1 in the supplementary information (SI).

### Particle size distribution

Particle size distributions (PSDs) of the biological reference materials in the different suspension reagents were measured using laser diffraction particle size analyzer (Mastersizer 3000, Malvern Panalytical, Malvern, UK). Sample suspensions were prepared as stated above with a concentration of 10 mg mL^−1^. To approx. 100 mL of deionized water, between 0.5 and 1.5 mL of the respective suspension were added in the Hydro 2000S-A accessory (Malvern Panalytical, Malvern, UK) to achieve an obscuration between 2 and 10%. The stirrer was set to 3000 rpm and each sample was measured in three cycles of each 45 s at 633 nm and one cycle of 15 s at 470 nm. After each sample, the measurement cell was rinsed three times with deionized water and a new background was taken. The refractive indices were set to 1.39 for NIST 1577c and ERM-BB186, 1.52 for NIST 1486 [32], and 1.33 for the suspension reagents based on the high dilution factor with water.

### Instrumentation for TXRF measurements

For TXRF measurements, the following method parameters and instrumentation were used. S2 Picofox (Bruker Nano GmbH, Berlin, Germany) equipped with a molybdenum X-ray tube and a high-efficiency module operated at 50 kV voltage and 600 µA current was used. Software used for measurements and evaluation was Spectra 7.8.2.0 (Bruker Nano GmbH, Berlin, Germany). Background calculation was carried out with 40 cycles based on escape peaks and an optimized profile Bayes fit with a step width of 1 and a maximum number of stripping cycles of 100 for deconvolution. The background in the case is defined as the area below the baseline modeled by the given software parameters. In the case of Fe K_α_ at 6.41 keV, this refers to a region of interest from approx. 6.15 keV to 6.65 keV. Measurement time was set to 1000 s live time. Measurements were carried out under nitrogen atmosphere (5.0, MTI, Neu-Ulm, Germany) to minimize argon signal from air and interaction of remaining reagent traces (acidic vapor) with the instrument (e.g., detector window), especially in the case of concentrated nitric acid.

### Calibration

Titanium and chromium standard solutions used as internal standards (IS) for TXRF measurements were prepared from 1000 mg L^−1^ stock standards (Ti: Merck KGaA, Darmstadt, Germany; in 5 wt.-% HNO_3_, 0.5% HF; Cr: Merck KGaA, Darmstadt, Germany; in 0.5 mol L^−1^ HNO_3_) by dilution in 5 wt.-% nitric acid. Titanium was selected as IS for SRM 1577c and SRM BB186 due to its absence in the samples, which has been confirmed by the absence of any peak at 4.51 keV (see Figure S2 in the SI). For NIST 1486, the presence of Ba in the sample leads to a line overlap of Ba-L_α_ with Ti-K_α_. Consequently, Cr was selected as IS, as no peak is observed at 5.42 keV. The selected IS energy lines lie within the same spectral range as the elements of interest, primarily Fe. All spectra of sample digests can be found in Figure S2 in the SI. In each sample suspension run, one blank solution of the suspension reagent was included to check for contamination and used for blank correction of measurements. Quantitative evaluation was achieved using the IS and correlating its concentration with respect to the relative sensitivities to the net intensities found for the analyte and the IS as given by the following equation:

1 $${c}_{A}=\frac{{N}_{A}}{{S}_{A}}*\frac{{S}_{IS}}{{N}_{IS}}*{c}_{IS}$$

with net intensity N, relative sensitivity S, and concentration c with indices IS for internal standard and A for analyte.

Limits of detection and quantification were calculated by the following equations.

2 $$LOD=\frac{3 {c}_{i}\sqrt{{N}_{BG}}}{{N}_{i}}$$

3 $$LOQ=\frac{10 {c}_{i}\sqrt{{N}_{BG}}}{{N}_{i}}$$

with background intensity ${N}_{BG}$ and peak intensity ${N}_{i}$. Only elements fulfilling the LOQ criterion were included in the evaluation.

To evaluate the sensitivity, signal-to-noise ratios were calculated using the following ratio

4 $$SNR=\frac{{N}_{i}}{\sqrt{{N}_{BG}}}$$

Uncertainty of mass fractions of reference materials is the expanded uncertainty with a coverage factor k = 2 corresponding to a level of confidence of about 95% [33, 34].

5 $$U=2 \frac{ SD}{\sqrt{n}}$$

### Cleaning procedures

All the necessary procedures to avoid and/or minimize sample contamination according to trace metal analysis protocols were applied as described in detail in *Gruber *et al. [26]. In brief, sample and solution preparation steps were carried out in a class 100 laminar flow hood (SuSi Super Silent, Spectec GmbH, Erding, Germany). Quartz glass sample carriers were first wiped using cleanroom wipes (Spec-Wipe 3, VWR International GmbH; Darmstadt, Germany) soaked with ultrapure water and then placed for 2 h in 10% Hellmanex III solution (Hellma Analytics GmbH & Co KG, Mühlheim, Germany) at 80 °C. Thereafter, they were rinsed three times with UPW and placed in a 10% nitric acid bath for 2 h at 80 °C. Finally, after rinsing three times with UPW again, the carriers were placed in a drying oven for 2 h at 120 °C. Cooled carriers were coated with 10 μL silicone solution (in 2-propanol, Serva electrophoresis GmbH, Heidelberg, Germany) and checked for purity by TXRF measurement (measurement time 100 s, 50 kV, 600 μA) prior to use. All consumables like tips and tubes were cleaned for at least 24 h in 10% nitric acid baths and stored until use in 0.5% nitric acid. Prior to use, each item was rinsed three times with UPW. All reagents, containers, and sample carriers used in this study were checked for blank values, and appropriate measures were taken when contamination was detected.

## Results and discussion

### Sample homogeneity and sample amount

Sample preparation by means of suspension using TXRF is a quasi-direct microanalysis method. Thereby, the homogeneity of the sample is crucial for defining a minimum sample weight that still allows for a representative analysis. Therefore, as a first step, sample homogeneity and minimum required sample amount were evaluated. To this end, the suspension procedure reported in [25, 26] was used to analyze replicates of 20 mg of the CRMs bovine liver (SRM 1577c) and pig kidney (ERM186), as this method had been successfully validated previously [35]. The obtained data was used to calculate element-specific homogeneity constants (H_E_) according to *Kurfürst *et al*.* [36] and these were put into perspective with *Horwitz* relative standard deviation (RSD_H_) [37]. Then, the minimum sample amount (m_min_) was calculated according to Eq. 6 from RSD_H_, H_E_, and the pooled relative standard deviation (RSD_P_). Desired RSD should range between 50 and 200% of RSD_H_; therefore, it is expressed as two times the RSD_H_. Results and formulas for calculating H_E_, RSD_H_, and RSD_p_ can be found in the SI.

6 $$m_{min}=\left(\frac{H_E}{\sqrt{\left(2\ast RSD_H\right)^2-RSD_p^2}}\right)^2$$

As a result, calculated minimal sample amounts to achieve a desired RSD are for all elements of interest below 3 mg (s. Table 2). This is in agreement with the information and results of homogeneity studies by solid sampling electro thermal atomic absorption spectrometry (SS-ET-AAS) [38–40]. These showed homogenous distribution for minor and trace elements for a sample amount between 20 µg and 70 µg of SRM 1577c. For ERM-BB186, elemental distribution was deemed homogenous except for Cr and Ni, which were not evaluated in the present study. In conclusion, the sample weight is not a limiting factor in this investigation as considering the sensitivity of TXRF measurements and the analyte concentrations involved, sample weights exceeding 3 mg are appropriate. Such small sample sizes allow for microanalysis, which is particularly important in biomedical research involving animal experiments, where the quantity of available samples may be inherently limited. For example, bone specimens (radius and ulna) from mouse models weigh only a few milligrams per bone [41] and extensive separation procedures yield only small numbers of isolated single-cell types [42].

**Table 2:** Element-specific minimal sample amounts (m_min_) to achieve desired RSD

Reference material	Element	Minimal sample amount m_min_(mg)
NIST 1577c	Se	0.63*
	Mn	0.38
	Ca	1.32
	Zn	1.96
	Fe	0.22
	Cu	2.03
ERM-BB186	Mn	0.91*
	Se	0.98*
	Cu	0.001
	Zn	1.28
	Fe	0.34

*In these cases, values are calculated from an estimated relative sampling error (RSE) of 5% as pooled RSD_P_^2^ exceeded overall RSD^2^; RSE for other elements were determined to be < 5%; further details, see SI

However, in the application to (other) real-world samples, it must be considered that the minimum required sample amount may differ from the one calculated here, as H_E_ depends strongly on the sample preparation and the intrinsic properties of the sample being analyzed. Both the particle size following sample grinding and changes in the PSD during suspension preparation have a significant influence on the required minimum sample amount. Achieving adequate homogeneity for microanalysis may not always be possible because the higher the residual sample heterogeneity, the larger the amount of material needed to obtain representative and reproducible results. Consequently, sample amounts of 10 mg and 20 mg were employed to evaluate the performance of the different suspension reagents in this study. The effects of PSD on SA-TXRF are discussed in detail in the following section.

### Particle size distributions and sample type classification

Matrix effects are typically considered negligible in TXRF due to the infinitesimally thin sample layers, approximately 50 nm for mineral powders and 4 µm for organic tissues [43], deposited on the reflective carrier. However, this assumption may no longer hold when using suspension-assisted sample preparation. The three reference materials used in this study are supplied as fine powders with particle size distributions predominantly below 7.57 µm for bovine liver (SRM 1577c [38]), below 250 µm for pig kidney (ERM‑186 [39]), and below 355 µm for bone meal (SRM NIST 1486 [44]). These particle sizes exceed the above-given ideal sample thicknesses for thin layers, below which negligible matrix effects are expected. However, these criteria are less applicable to suspensions of biological powders, since achieving a homogeneous layer is improbable. When investigating such suspensions, providing a constant intensity ratio of analyte and internal standard through homogeneous distribution of the internal standard over the height of the sample particles results in significantly higher practical upper thickness limits [12]. In this context, *Fernández-Ruiz* et al*.* [24] showed the effect of PSD on analytical performance finding that lower average sizes, as well as minimizing the width of particle size distributions, resulted in higher accuracy and lower detection limits in SA-TXRF analysis. In agreement with this, a previous study by the authors on TXRF trace element determination in placenta tissue showed that enhanced grinding and finer comminution significantly improved agreement between results obtained using SA-TXRF and those from digestion-based TXRF analysis [25].

In the present study, ideally homogenized materials in the form of CRMs were used and therefore no further grinding experiments were performed. Nevertheless, suspension reagents can promote the partial decomposition of the organic matrix and the dissolution of mineral components and analytes, thereby affecting the actual PSD. Accordingly, the actual PSDs of the three CRMs in the five different suspension media tested herein were investigated using laser diffraction particle size analysis.

**Fig. 1:**
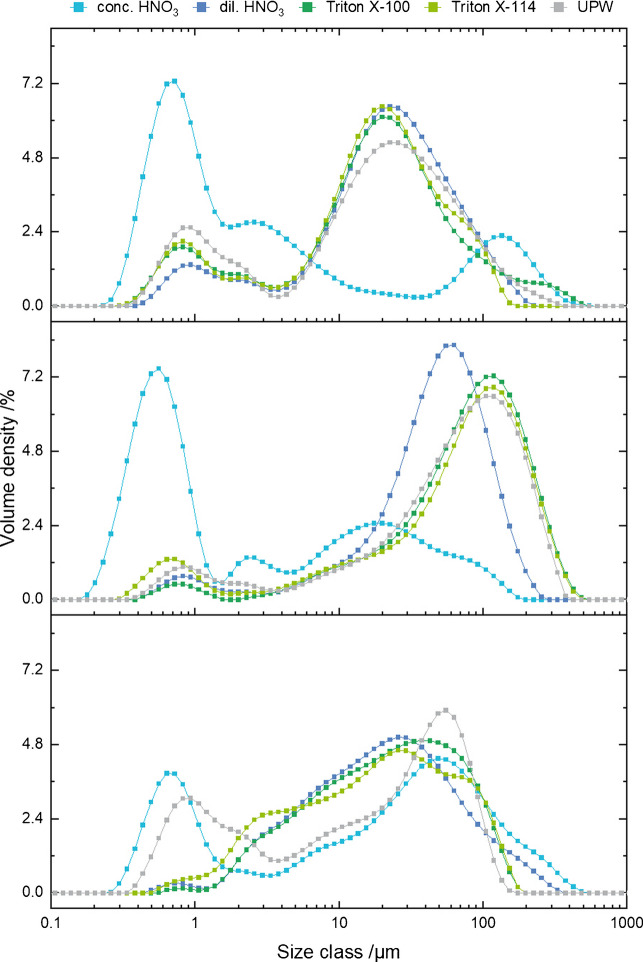
Particle size distributions of suspensions of **a** bovine liver (NIST 1577c), **b** pig kidney (ERM-186), and **c** bone meal (NIST 1486) in the five different suspension media, namely concentrated nitric acid (light blue), dilute nitric acid (dark blue), Triton X-100 (dark green), Triton X-114 (light green), and ultrapure water (gray)

The digestive effect of highly concentrated, reactive nitric acid leads to a significant reduction in the particle size of all three CRMs (s. Figure 1). In the other suspension media, very similar PSDs are obtained, especially for the soft biological matrices of bovine liver (s. Figure 1a) and pig kidney (s. Figure 1b), while for bone meal (s. Figure 1c), a reduced PSD is also observed when water is used as the suspension medium.

**Fig. 2:**
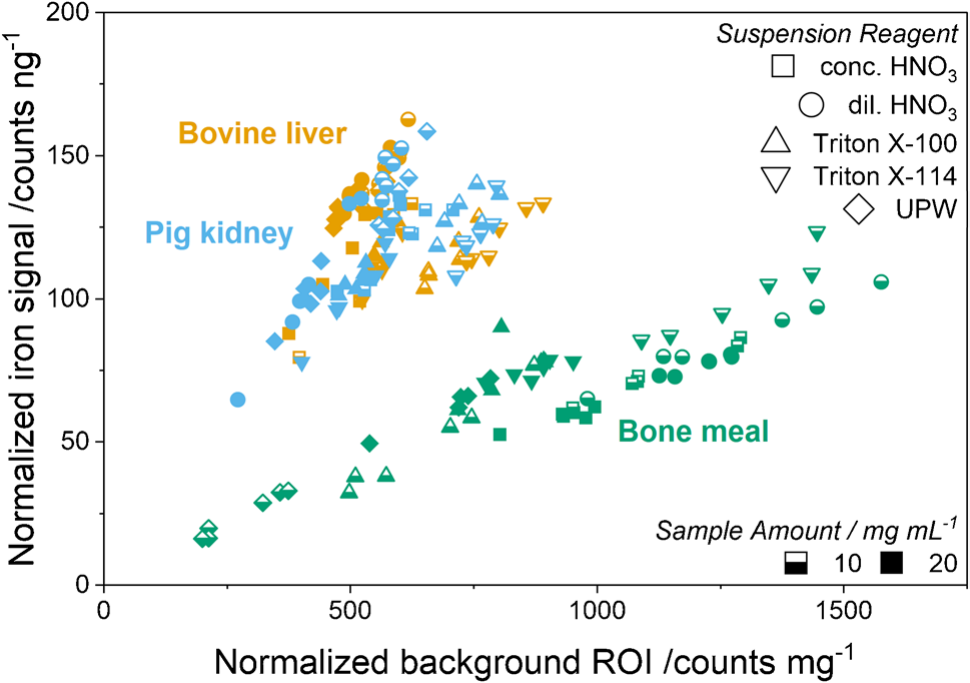
Comparison of normalized iron (Fe K_α_: 6.045 keV) signals, as an exemplary analyte, in relation to the background in the region of interest (ROI) for the five different suspension reagents and two tested sample amounts. (The Fe signals are normalized to the sample amount and the iron mass fraction of the respective reference material. The background signals are normalized to the sample amount. Each data point corresponds to an individually prepared sample carrier. Measurement time: 1000 s)

As to be expected, the two soft biological reference materials, bovine liver and pig kidney, show comparable spectral characteristics (s. Figure 2), indicating a similar matrix composition and elemental distribution. Their higher signal intensities and lower background levels relative to the hard biological material, bone meal, result from a more favorable excitation and detection of Fe in the soft, dark matter matrices. A similar pattern is found for zinc, as shown in Figure S4 in the SI.

In addition to particle size, the amount of sample deposited on the reflector is therefore a key parameter, as it affects both the effective layer thickness and the morphology of the dried residues. An increase in sample mass from 10 mg (half-filled symbols in Fig. 2) to 20 mg (filled symbols in Fig. 2) leads to higher Fe signals and increased background intensities, consistent with the expected scaling of analyte mass and matrix effects. After normalizing the background ROI and the analyte signal to the sample mass, a comparison of both sample amounts shows clear clustering in the two soft matrices, but less so in the hard matrix. A much lower increase in Fe signal compared to background signal is found in the hard matrices with a larger spread of the data for the lower sample amount.

Notably, the distinct clustering observed for the different suspension reagents for each biomaterial highlights their pronounced influence on sample dispersion. Wettability of the reagents on TXRF sample carriers determines the droplet size and resulting residue area. As carrier surfaces are treated with silicone to promote droplet formation and limit spreading, more polar reagents yield smaller droplets and higher sample concentration per unit area (see Figure S9 in the SI). Among the investigated reagents, Triton solutions thus form larger droplets than UPW or nitric acid, resulting in lower areal sample density and altered deposition morphology. In addition, reagent reactivity and suspension stability further contribute to the observed clustering of suspension reagents in Fig. 2. As discussed above, acidic suspension media, such as concentrated and dilute nitric acid (square and circle symbols in Fig. 2), promote partial decomposition of the organic matrix, dissolution of mineral components, and increased analyte solubility resulting in the case of concentrated nitric acid in a significant reduction in particle size (s. Figure 1). This improves the homogeneity of sample residue and internal standard deposited on the sample carrier and helps approximate the ideal thin-film condition in TXRF. In contrast, ultrapure water (UPW; diamond symbols in Fig. 2) exhibits low reactivity and poor solubilizing capacity for organic and mineral matrices such as hydroxyapatite, which compromises suspension stability and impedes homogeneous application onto the sample carrier. Accordingly, lower Fe signals and background intensities for all three CRMs result. The addition of surfactants such as Triton enhances dispersion formation and stability, yielding intermediate signals and background levels between those obtained with nitric acid media and UPW.

### Recovery

Evaluation focuses on elements in the trace and microelement concentration range (below 500 mg kg^−1^), which are particularly of interest in biomedical and biological research and are certified in the respective matrix reference materials (s. Table 1). In this context, the primary objective was to assess the trueness of element quantification when employing different suspension reagents. As anticipated, the two soft biological matrices, pig kidney and bovine liver, showed comparable behavior, whereas bone meal, representing a hard biological matrix, deviated from this trend.

**Fig. 3:**
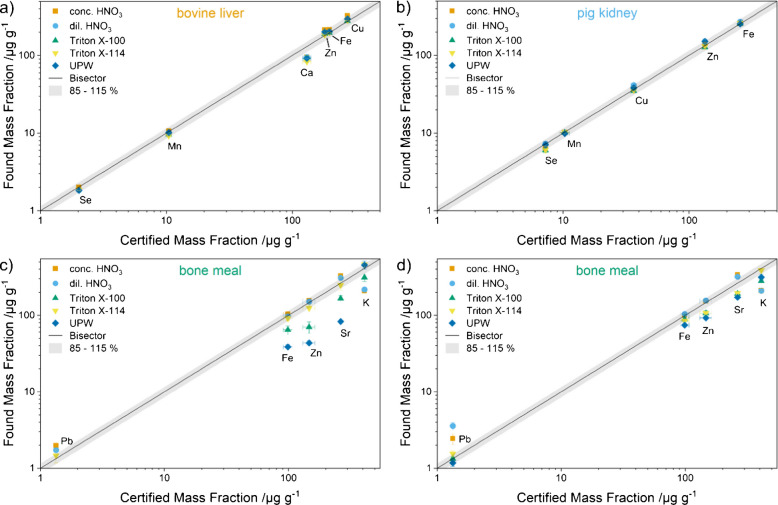
Recovery of mass fractions in different CRMs found by SA-TXRF using different suspension reagents. **a** NIST 1577c; **b** ERM-BB186; **c**, **d** NIST 1486. (Data are mean values ± expanded uncertainty with k = 2; n = 6; measurement time 1000 s; sample weight: 10 mg, except **d** 20 mg; suspension volume: 1 mL)

For bovine liver and pig kidney, recoveries generally fall within an acceptable range from 85 to 115% (with the exception of Ca in bovine liver) independent of the applied suspension reagent, sample weight and element concentration (s. Figures 3a, b and S5a, b in SI). For bovine liver, this spans over a broad mass fraction range from 2.031 mg kg^−1^ for the trace element Se to 275 mg kg^−1^ for Cu. In pig kidney, Se mass fraction is certified as 10.3 mg kg^−1^ and Fe as 255 mg kg^−1^. A notable exception is calcium (Ca) in bovine liver, for which lower recoveries in the range between 65 and 75% were obtained across all suspension reagents. A possible explanation is its relatively low atomic number in conjunction with a potassium-rich sample matrix with around 10.000 mg kg^−1^, complicating deconvolution as Ca K_α_ (3.692 keV) and K K_α_ (3.314 keV) peaks are in close proximity.

Bone meal shows more pronounced differences between suspension reagents, with recovery values ranging from 30 to 120% (s. Figure 3c, d). Due to its different chemical composition characterized by approximately 25 wt.-% Ca and 12 wt.-% P, the influence of different suspension reagents is much more prominent. Diluted or concentrated nitric acid yielded the best agreement with certified values (recoveries ranging from 97 to 129%), except for K (present at 400 mg kg^−1^), where ultrapure water and Triton X-114 performed better. The spectral overlap between Ca and K likely complicates accurate K quantification, with acidic reagents elevating Ca signals and thereby biasing further K determination. Moreover, significantly lower recoveries for Fe, Zn, and Sr were obtained with ultrapure water and Triton X-100 when using only 10 mg sample mass instead of 20 mg. Conversely, the slight overestimation of Pb at 10 mg became markedly more pronounced at 20 mg with diluted or concentrated nitric acid. No definitive explanation exists beyond the possibility of a random outlier or contamination event.

### Precision

In a second step, the precision of the obtained results depending on suspension reagents was evaluated by determining relative standard deviation (RSD). No systematic overall trend was observed across matrices, suspension agents, or element concentrations. Variability was primarily driven by differences between the three individual sample carriers, and these carrier-specific effects were directly reflected in the magnitude of the RSD. In this regard, it is notable that blank values are often found for Zn, which is a common contaminant in many materials and reagents.

**Fig. 4:**
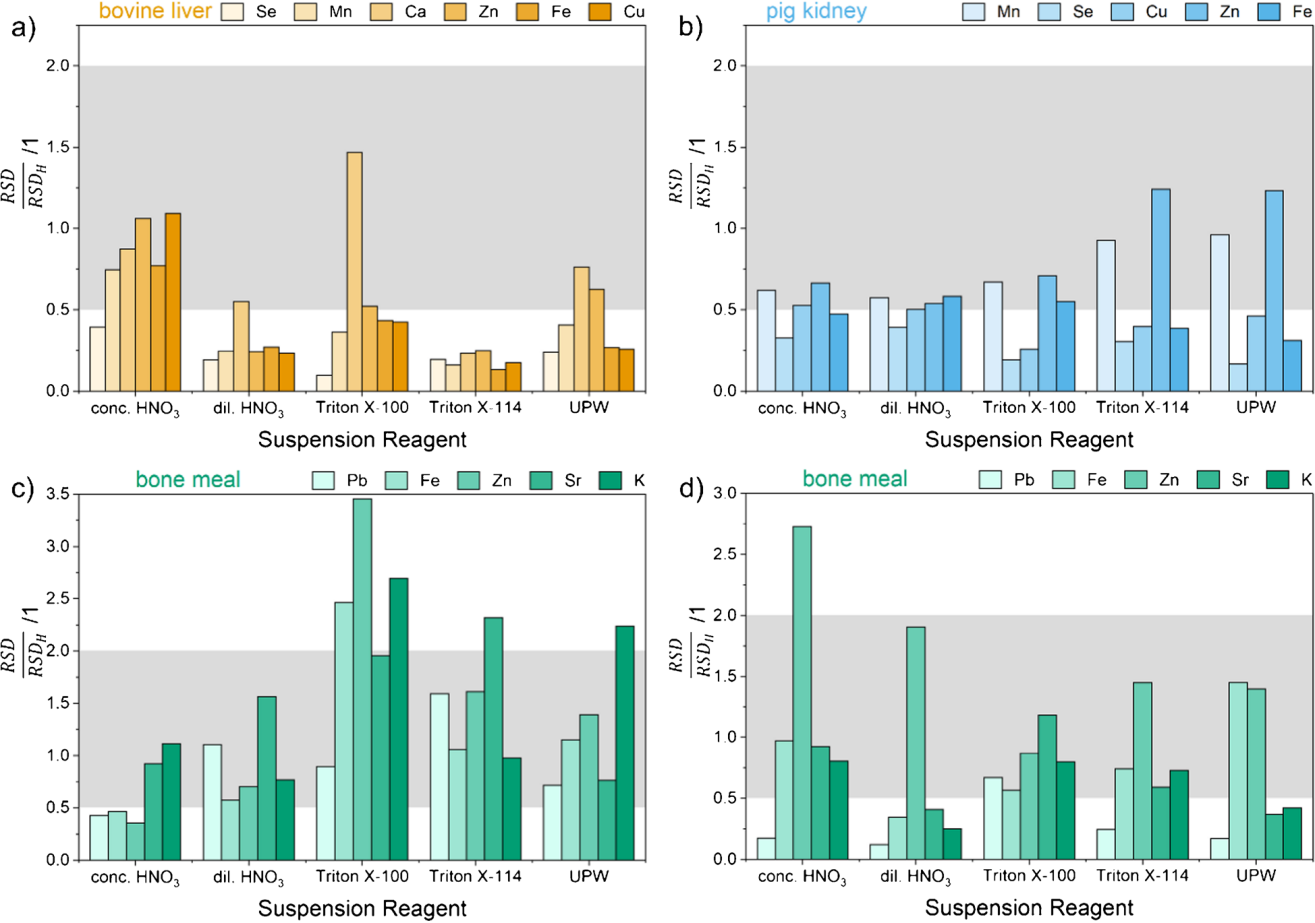
Ratio of observed RSD to RSD_H_ for **a** NIST 1577c; **b** ERM-BB186; **c**, **d** NIST 1486. (sample weight: 10 mg, except **d** 20 mg; suspension volume: 1 mL)

The observed relative RSDs were correlated with the RSD_H_ and their ratio is presented in Fig. 4, indicating generally acceptable precision. Exceptions with undesirable RSD values exceeding 200% of RSD_H_ occurred only for bone meal using 10 mg sample mass and Triton X-100, but these were largely mitigated by increasing the sample mass to 20 mg.

### Signal-to-noise ratios

**Fig. 5:**
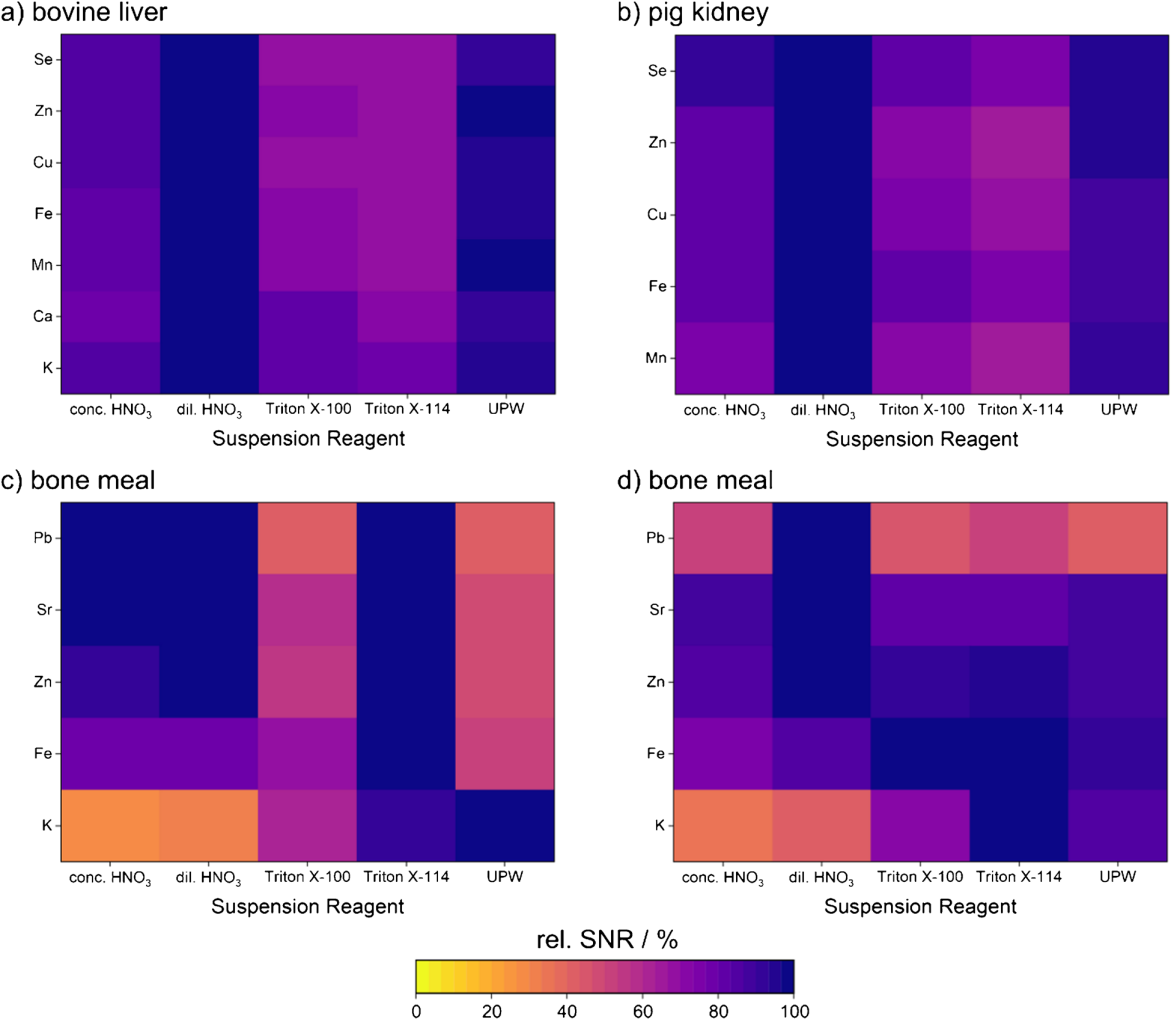
Relative signal-to-noise ratios (rel. SNRs) normalized to the maximum value observed for each element (set to 100%) for **a** NIST 1577c; **b** ERM-BB186; **c**, **d** NIST 1486 (sample weight: 10 mg, except **d** 20 mg; suspension volume: 1 mL)

The above-presented results have shown that suspension-assisted does not provide ideal conditions for TXRF analysis, meaning that matrix effects should be considered. One approach to quantify these effects is calculating signal-to-noise ratios (SNRs, s. Eq. 4), which were normalized to the maximum value observed for each element (set to 100%) to enable comparison of different concentration levels. In contrast to the recoveries and standard deviations, the SNRs display a clear dependence on the applied suspension reagent (s. Figure 5) with the highest values typically obtained using either diluted nitric acid or ultrapure water. Looking at the results more closely, the high SNRs in ultrapure water are mainly due to lower background, while high SNRs in diluted nitric acid are the result of higher analyte signals. This is consistent with the absence of partial matrix digestion in ultrapure water.

In bovine liver and pig kidney, higher sample weight increases the SNRs without altering the trends observed between suspension reagents (s. Figure S7 in SI). Moreover, higher-Z elements exhibit systematically greater differences between the two non-ionic surfactants (Triton X-100 and Triton X-114). These yield significantly lower SNRs than acidic or aqueous media because of the above-described differences in wettability and resulting larger areal sample densities. For bone meal, this effect is less pronounced due to the higher mean atomic number of the matrix which results in a higher background. Nevertheless, different patterns are visible for the two tested sample weights, though highest SNRs are generally obtained for Triton X-114 followed by nitric acids for low sample weights and ultrapure water for higher sample weights with a strong element-dependent behavior in both concentrations.

The element-specific sensitivity is directly related to the SNR and was calculated for all studied elements as the lower limit of quantification (LOQ; see Tables S2–S4 in SI for details). The obtained values for the recommended methods are given below in the corresponding section.

### Greenness evaluation

**Fig. 6:**
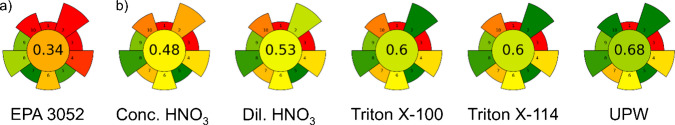
Calculated AGREEprep scores and pictograms for **a** microwave-assisted sample digestion adapted from EPA 3052; and **b** suspension-assisted sample preparation of bovine liver and pig kidney using different reagents. (Sample weight: 10 mg, sample volume: 1 mL; further details used for calculation can be found in the SI)

To integrate sustainability considerations into the evaluation of suspension reagents, AGREEprep scores were calculated and compared with those for the microwave-assisted digestion procedure adapted from EPA Method 3052 [22]. AGREEprep is a greenness metric focused on sample preparation independent of the measurement technique. It quantifies greenness on a score from 1, for the most green, and 0, for the least green approach, based on the 10 principles of Green Sample Preparation enabling comparison of different sample preparation methods [16]. Details and parameters used for calculation of AGREEprep scores can be found in Tables S5 and S6 in the SI. The score calculated for the microwave-assisted digestion procedure was 0.34 while suspension-assisted sample preparation procedures scored a minimum of 0.48. The primary differentiating criteria resulting from the selection of suspension reagents are the amounts of hazardous materials (criterion 2) and the operator safety (criterion 10). For pig kidney and bovine liver, the obtained AGREEprep scores are identical as the only difference in the experimental workflow was the choice of suspension reagent (s. Figure 6). For bone meal, slightly higher scores resulted from reduced preparation time, yielding greater sample throughput (criterion 6) and lower energy consumption (criterion 8; s. Figure S8 in the SI).

Ultrapure water is obviously the greenest suspension reagent, owing to the absence of hazardous substances and maximal operator safety, yielding an AGREEprep score of 0.68. Both nitric acid solutions and Triton surfactants carry three listed hazards each, but differing volumes result in distinct scores: diluted HNO_3_ (0.53) outperforms concentrated HNO_3_ (0.48), while Triton X-100 and Triton X-114 both achieve 0.60, ranking second overall. This unexpectedly high performance of the surfactants, despite their classification as substances of very high concern and restriction under European Chemicals Agency regulations [45], derives primarily from the minimal volumes employed, as AGREEprep does not explicitly penalize certain biological hazards such as aquatic toxicity or endocrine-disrupting potential.

Overall, the greenness evaluation demonstrates a clear advantage of suspension-assisted sample preparation over conventional microwave-assisted digestion, with ultrapure water being the most sustainable option within the present assessment framework. Furthermore, compared to the digestion method, all suspension approaches entail lower energy consumption (criterion 8), higher sample throughput (criterion 6), substantially reduced sample amounts (criterion 5), and hazardous materials (criterion 2), as well as less waste generation (criterion 4).

### Recommended methods

To decide on the optimum suspension reagent for sample preparation, the multi-criteria matrix in Table 3 compares the values achieved by SA-TXRF with defined target criteria. For this purpose, accuracy, sensitivity, and greenness were taken into account, with the precondition that trueness is given, i.e., the element recovery was between 85 and 115%. For precision, it was determined that RSD must be 10% or less. For sensitivity, calculated LOQ equivalents must be below the certified mass fraction. Regarding sustainability, we compare SA-TXRF with the adapted standard procedure involving microwave-assisted sample preparation. Therefore, any AGREE score higher than that of the reference method (0.34) is considered beneficial.

**Table 3:** Multi-criteria decision matrix, determined target values and achieved values for the three matrix CRMs investigated

(a) **Bovine liver (CRM 1577c; sample weight: 10 mg)**				
**Criteria**	Trueness*	**Precision (%)**	**LOQ (mg kg**^**−1**^**)**	**Greenness**
*Target value*	*Recovery: 85–115%*	*RSD* ≤ *10%*	*LOQ* < *certified mass fraction*	*AGREE score* > *0.34*
Conc. HNO_3_	**Fe, Mn, Se, Zn**	**Fe (6), Mn (8), Se (6), Zn (8)**	**Fe (0.59), Mn (0.73), Se (0.21), Zn (0.36)**	**0.48**
Dil. HNO_3_	**Cu, Fe, Mn, Se, Zn**	**Cu (2), Fe (2), Mn (3), Se (3), Zn (2)**	**Cu (0.35), Fe (0.53), Mn (0.66), Se (0.19), Zn (0.30)**	**0.53**
Triton X-100	**Cu, Fe, Mn, Zn**	**Cu (3), Fe (3), Mn (4), Zn (4)**	**Cu (0.45), Fe (0.68), Mn (0.84), Zn (0.39)**	**0.60**
Triton X-114	**Cu, Fe, Mn, Se, Zn**	**Cu (1), Fe (1), Mn (2), Se (2), Zn (2)**	**Cu (0.47), Fe (0.71), Mn (0.88), Se (0.27), Zn (0.41)**	**0.60**
UPW	**Cu, Fe, Mn, Se Zn**	**Cu (2), Fe (2), Mn (5), Se (3), Zn (5)**	**Cu (0.35), Fe (0.53), Mn (0.66), Se (0.19), Zn (0.30)**	**0.68**
(b) **Pig kidney (ERM186; Sample weight: 10 mg)**				
**Criteria**	Trueness*	**Precision**	**LOQ (mg kg**^**−1**^**)**	**Greenness**
*Target value*	*Recovery: 85–115%*	*RSD* ≤ *10%*	*LOQ* < *certified mass fraction*	*AGREE score* > *0.34*
Conc. HNO_3_	**Cu, Fe, Mn, Se, Zn**	**Cu (5), Fe (3), Mn (7), Se (4), Zn (5)**	**Cu (0.40), Fe (0.65), Mn (0.81), Se (0.23), Zn (0.37)**	**0.48**
Dil. HNO_3_	**Cu, Fe, Mn, Se, Zn**	**Cu (4), Fe (4), Mn (7), Se (4), Zn (4)**	**Cu (0.31), Fe (0.52), Mn (0.65), Se (0.19), Zn (0.28)**	**0.53**
Triton X-100	**Cu, Fe, Mn, Se, Zn**	**Cu (2), Fe (4), Mn (8), Se (2), Zn (5)**	**Cu (0.37), Fe (0.59), Mn (0.86), Se (0.23), Zn (0.35)**	**0.60**
Triton X-114	**Cu, Fe, Mn, Se, Zn**	**Cu (4), Fe (3),** Mn (11), **Se (4), Zn (9)**	**Cu (0.21), Fe (0.63), Mn (0.91), Se (0.28), Zn (0.35)**	**0.60**
UPW	**Cu, Fe, Mn, Se, Zn**	**Cu (4), Fe (2),** Mn (11), **Se (2), Zn (9)**	**Cu (0.32), Fe (0.52), Mn (0.64), Se (0.19), Zn (0.28)**	**0.68**
(c) **Bone meal (SRM NIST 1486; Sample weight: 20 mg)**				
**Criteria**	Trueness*	**Precision**	**LOQ (mg kg**^**−1**^**)**	**Greenness**
*Target value*	*Recovery: 85–115%*	*RSD* ≤ *10%*	*LOQ* < *certified mass fraction*	*AGREE score* > *0.34*
Conc. HNO_3_	**Fe, Zn**	**Fe (4), Zn (3)**	**Fe (1.1), Zn (0.5)**	**0.52**
Dil. HNO_3_	**Fe, Zn**	**Fe (2), Zn (2)**	**Fe (1.0), Zn (0.5)**	**0.57**
Triton X-100	**Fe, Pb**	Fe (19)**, Pb (7)**	**Fe (0.7), Pb (0.3)**	**0.63**
Triton X-114	**Fe, K, Pb**	**Fe (3), K (6),** Pb (11)	**Fe (0.7), K (13.0), Pb (0.3)**	**0.63**
UPW	**Pb**	Pb (11)	**Pb (0.2)**	**0.71**

*All listed elements meet the criteria of 85–115% recovery. All values in bold also meet the stated target value for precision, LOQ, and greenness

For both soft biological materials (bovine liver and pig kidney), it is apparent that diluted nitric acid and ultrapure water produce similar, good results. With only two exceptions, namely Mn showing a slightly higher RSD of 11.4%, and Se exhibiting low sensitivity in pig kidney tissue when using UPW, all criteria are met for the certified trace elements in these matrices. Therefore, we can recommend SA-TXRF using either water or diluted nitric acid for soft biological materials, as it provides a sustainable method with quantitative recovery, acceptable precision and sensitivity. The latter results in limits of detection (LOQs) that agree with the concentrations to be determined in biomedical research, e.g., 5 µg L^−1^ for iron (further values s. Tables S2–4 in the SI). Therefore, due to equivalent performance, the greener SA preparation using water as suspension reagent should be preferred.

As expected, the five suspension reagents performed significantly differently when investigating hard biological material, i.e., bone meal. None of the reagents allows for the accurate determination of all four certified elements, namely Fe, K, Zn, and Pb. Using Triton X-114, however, at least the three elements, Fe, K, and Pb, can be quantitatively recovered and all further criteria are met, except for a slightly higher RSD for Pb. The LOQ for Fe was found to be 7.6 µg L^−1^ for SA-TXRF using Triton X-114. Considering greenness using the above assessment framework, the proposed SA-TXRF method is beneficial over microwave-assisted sample preparation. However, Triton X-114 is classified as a substance of very high concern, and the high AGREEprep score primarily derives from the minimal volume employed here.

### Comparison to other TXRF methods

Table 4 compares recently published sample preparation strategies for trace element analysis in soft and hard biomedical tissues using TXRF. Across all cited studies, satisfactory analytical performance is reported in terms of trueness. However, clear methodological differences emerge when suspension-assisted approaches are compared with conventional digestion procedures. Microwave-assisted acid digestion remains the most widely used preparation technique for biomedical tissues. While it is well established due to its robustness and broad applicability, it has several disadvantages. These include the requirement for significantly larger sample amounts, ranging from 200 to 1430 mg compared to 10 mg to 100 mg for reported SA approaches. Consequently, digestion-based TXRF methods also require up to ten times higher volumes of mostly aggressive reagents and have extended preparation and run times. In contrast, suspension-assisted methods enable quasi-direct measurement with minimal chemical treatment, typically requiring only 1 mL of reagents and allowing for sample preparation times of up to a maximum of 30 min, while demonstrating good analytical performance.

**Table 4:** Studies reported since 2020 on the analysis of trace elements in biomedical tissue samples using TXRF and different sample preparation approaches. (Sorted by publication date, newest first; *n.a*, not applicable; suspension-assisted approaches marked in bold)

Sample matrix	Sample preparation				Internal standard	Applied sample volume	Reference measurement technique	Ref
	Procedure	Reagents	Duration, Temperature	Sample amount				
Bovine liver tissue (SRM 1577c), pig kidney tissue (ERM-BB 186), bone meal (SRM 1486)	**Suspension-assisted** (incl. sonication)	1 mL of either 65 wt.-% HNO_3_, 12 wt.-% HNO_3_, 1 vol.-% Triton X-100, 1 vol.-% Triton X114, or UPW	7–19 min, 40 °C	10–20 mg	Ti (25–50 µL of 100 mg L^−1^); or Cr (10 µl of 100 mg L^−1^)	10 µL	n.a	This work
Oyster tissue (NIST 1566b), bovine liver (NIST 1577c)	Acid digestion	2.5 mL of 65 wt.-% HNO_3_	8 h, 195 °C	200 mg	Ga (4–215 µL of 1 mg g^−1^)	6 µL	n.a	[17]
Murine liver tissue	Radioimmunoprecipitation assay buffer-based lyses (incl. sonification, incubation, centrifugation)	50 mM Tris, 150 mM NaCl, 2 M EDTA, 0.5% sodium deoxycholate, 0.1% sodium dodecyl sulphate, 1% NP-40 containing 1 mg mL^−1^ protease inhibitor	> 25 min, 4 °C	25 mg	Y (1 mg L^−1^)	10 µL	ICP-MS/MS (after microwave digestion)	[46]
Rat tissues of kidney, rat heart, spleen, lung	Microwave-assisted acid digestion	65 wt.-% HNO_3_, 2.5 mL per 1 g of tissue	45 min, < 190 °C < 870 W	377–1430 mg	Ga (100 µL of 1000 mg L^−1^)	5–6 µL	ICP-OES, ICP-MS	[18]
Human tissues of liver, stomach, aorta, bladder, heart, uterus, skin, muscle, lung, small intestine, ascending colon	Microwave-assisted acid digestion	5 mL of 65 wt.-% HNO_3_, 1 mL of 30 wt.-% H_2_O_2_	60 min, max. 170 °C	200 mg	Ga (final conc. 10 mg L^−1^)	10 µL	ICP-OES	[35]
	**Suspension-assisted** (after optionally cryomilling)	1 mL of 2 M HNO_3_	15 min	20 mg	Ga (10 μg mL^−1^)		n.a.	
Human placenta	Microwave-assisted acid digestion	8 mL of 65 wt.-% HNO_3_, 2 mL of 30 wt.-% H_2_O_2_	40 min, max. 200 °C	250 mg	V (50 μL of 100 mg L^−1^)	10 µL	ICP-OES	[25]
	**Suspension-assisted**	1 mL of 65 wt.-% HNO_3_	30 min, 40 °C	10 mg				
Mussel muscle (GBW08571)	**Suspension-assisted** (incl. sonication)	1 g of 1% (w/v) Triton X-100	10–15 min	20–50 mg	Y (final conc. 10 mg kg^−1^)	5 µL	ICP-OES	[28]
Mouse liver tissue	**Suspension-assisted** (incl. sonication)	1 mL of 65 wt.-% HNO_3_	17 min, 40 °C	3–30 mg	Ti (10 μL of 100 mg L^−1^)	10 µL	GFAAS	[26]
Bovine liver tissue (SRM 1577c)		4 mL of 65 wt.-% HNO_3_	19 min, 40 °C	100 mg				

## Conclusion

Suspension-assisted TXRF is a valid alternative to microwave-assisted digestion for biological and biomedical samples. However, significant differences occur in the achieved analytical performance between soft and hard biomaterials. This is due to the nature of the chemical composition and physical properties of the different samples, in conjunction with the principles of total reflection X-ray fluorescence analysis. Particularly clear differences in the selection of various suspension reagents were observed with the hard biomaterial, bone meal. Here, recoveries varied between 30 and 120% while for the soft biomaterials, recoveries were all above 65% and below 115%. Nevertheless, SA-TXRF methods can be found that allow for the accurate determination of traces and microelements in both sample types. Precision evaluated by RSD indicated generally acceptable values that were mostly far below 200% of RSD_H_. Observed variability reflected mainly differences between individually prepared sample carriers. In the case of bone meal, it was apparent that higher sample weight favors clearly lower RSD. Sensitivity of the recommended methods indicates the suitability of SA-TXRF for application in biomedical research with LOQs that are in the range of interest for the relevant elements. At the same time, all suspension-assisted approaches showed a significant superiority over microwave-assisted sample preparation in terms of methodological sustainability. Significantly lower reagent consumption, preparation time, and sample mass are required in comparison to digestion-based TXRF approaches. Calculated AGREEprep scores were significantly lower than that of comparable microwave-assisted TXRF analysis. Obviously, ultrapure water proved to be the most sustainable option, whereas Triton-based agents scored surprisingly well owing to the small volumes required, despite their known environmental concerns. Taken together, the results show that diluted nitric acid and ultrapure water provide the most balanced compromise between analytical performance and greenness with regard to selection of suspension reagent. Overall, the study demonstrates that carefully selected suspension reagents enable valid multi-element SA-TXRF analysis with significantly reduced environmental impact, supporting its broader adoption in routine trace and microelement determination.

## Supplementary Information

Below is the link to the electronic supplementary material.

Supplementary file1 (DOCX 1.78 MB)

## Data Availability

The data that support the findings of this study are openly available at https://zenodo.org under https://doi.org/10.5281/zenodo.17983954.

## Declarations

### Source of biological materials

Three biological reference materials were purchased from National Institute of Standards & Technology, Gaithersburg, MD, USA (SRM NIST 1577c: bovine liver; SRM NIST 1486: bone meal) and Institute for Reference Materials and Measurements, Geel, Belgium (ERM-BB186: pig kidney).
