# Supplementary material for: Evaluation of reagents for suspension-assisted total reflection X-ray fluorescence element analysis in biomedical samples
Source: Anal Bioanal Chem. 2026 Apr 30;418(16):5267–81. doi: 10.1007/s00216-026-06505-2 (PMC13424235; doi:10.1007/s00216-026-06505-2)
Supplement: Supplementary file 1 — Supplementary file1 (DOCX 1.78 MB) [file 216_2026_6505_MOESM1_ESM.docx]

**Supplementary Information**

# **Evaluation of reagents for suspension-assisted total reflection X-ray fluorescence element analysis in biomedical samples**

Sebastian Hauser^1)^, Marit Veit^1)^, Kerstin Leopold^1)*^

1) Institute of Analytical and Bioanalytical Chemistry (IABC), Ulm University, Albert‑Einstein‑Allee 11, Ulm 89081, Germany

* Corresponding author

Email: [kerstin.leopold@uni-ulm.de](mailto:kerstin.leopold@uni-ulm.de)

## Equations

Homogeneity constant H_E_

| $H_{E}=RSE*\sqrt{m}$ | (S1) |
| --- | --- |

Relative sampling error RSE

| $RSE^{2}=RSD^{2}-RSD_{p}^{2}$ | (S2) |
| --- | --- |

Horwitz RSD

| $RSD_{H}=2^{(1-0,5*\log_{10} \omega)}$ | (S3) |
| --- | --- |

Pooled standard deviation s_p_:

| $s_{p}=\sqrt{\frac{1}{\Sigma{(n}_{i}-1)}\sum\left( n_{i}-1 \right)s_{i}^{2}}$ | (S4) |
| --- | --- |

Relative SNR

| $rel. SNR_{i}=100 \%*\frac{SNR_{i}}{\max SNR_{i}}$ | (S5) |
| --- | --- |

## Sample preparation procedure


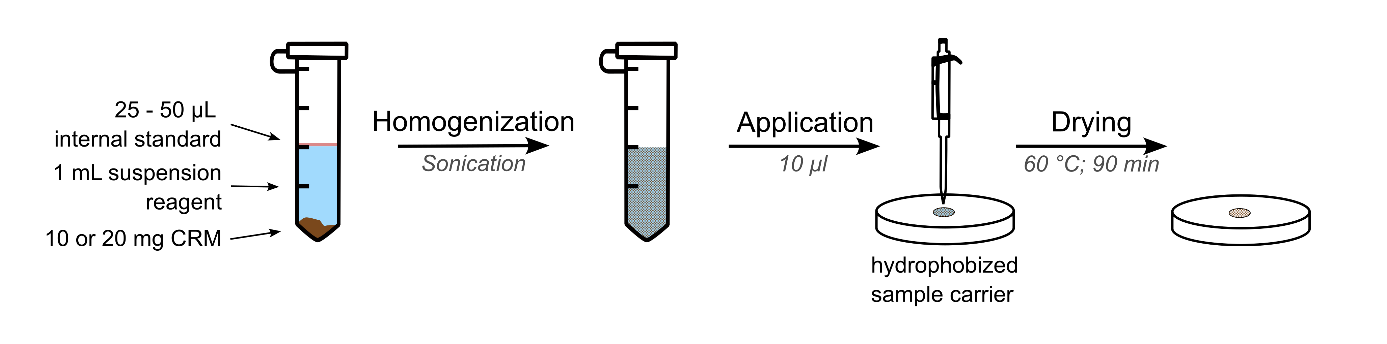


Figure S1: Schematic representation of the sample preparation procedure of SA-TXRF method.

## Spectra


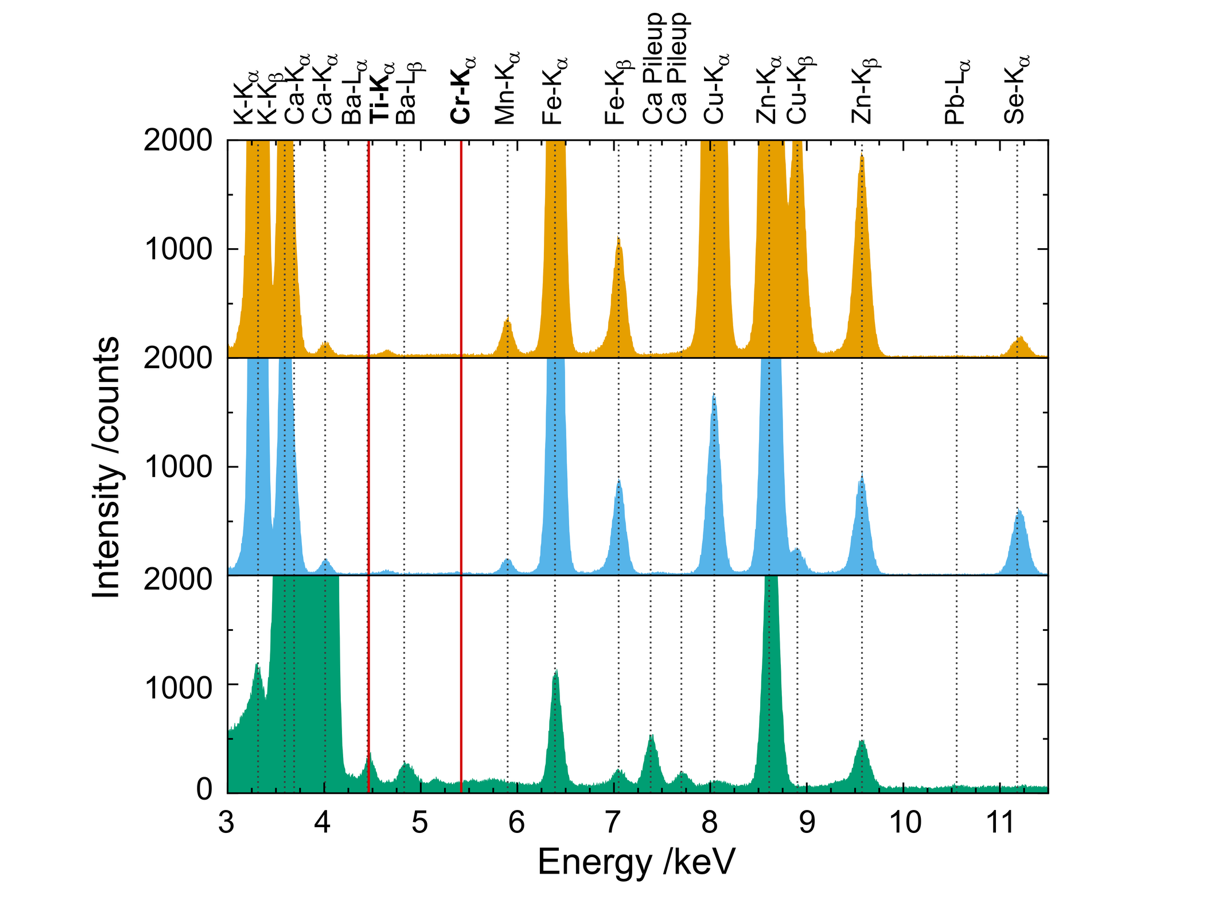


Figure S2: Exemplary qualitative TXRF spectra of CRMs digests without addition of internal standard with a measurement time of 1,000 s. The dashed lines indicate the energy lines of elements detected in the digests; Red lines indicate K_α_ lines of Ti and Cr which were selected as internal standards in the SA-TXRF method.

## Homogeneity

Table S1: Calculated element-specific relative sampling error (RSE), homogeneity constant (H_E_) and resulting RSD for a sample amount of 10 mg. (* In these cases, values are calculated from an estimated RSE of 5% as pooled RSDP² exceeded overall RSD².)

| CRM | Element | ω  /mgkg^‑1^ | RSD_H_  /% | RSD  /% | RSD_P_  / % | RSE  /% | H_E_  /%mg^‑1/2^ | RSE_10mg_  /% | RSD_10mg_  /% |
| --- | --- | --- | --- | --- | --- | --- | --- | --- | --- |
| NIST 1577c | Se | 2.031 | 14.38 | 3.20 | 3.26 | - | 22.61* | 7.15* | 7.86* |
|  | Mn | 10.46 | 11.24 | 6.94 | 6.29 | 2.95 | 13.21 | 4.18 | 7.57 |
|  | Ca | 131 | 7.68 | 6.88 | 5.86 | 3.61 | 16.15 | 5.11 | 1.32 |
|  | Zn | 181 | 7.32 | 7.00 | 5.61 | 4.18 | 18.70 | 5.91 | 8.20 |
|  | Fe | 197 | 7.22 | 3.95 | 3.67 | 1.45 | 6.49 | 2.05 | 4.21 |
|  | Cu | 275 | 6.87 | 6.83 | 5.57 | 3.95 | 17.69 | 5.59 | 7.94 |
| ERM-BB186 | Mn | 7.26 | 11.87 | 3.77 | 4.08 | - | 22.27* | 7.04* | 8.14* |
|  | Se | 10.3 | 11.26 | 1.79 | 1.86 | - | 22.27* | 7.04* | 7.28* |
|  | Cu | 36.5 | 9.31 | 2.77 | 2.76 | 0.14 | 0.608 | 0.192 | 2.77 |
|  | Zn | 134 | 7.66 | 4.79 | 2.88 | 3.82 | 17.10 | 5.41 | 6.11 |
|  | Fe | 255 | 6.95 | 2.64 | 1.94 | 1.79 | 8.01 | 2.53 | 3.18 |
| *: Values calculated from an estimated RSE = 5% ; n=5x3=15. | | | | | | | | | |


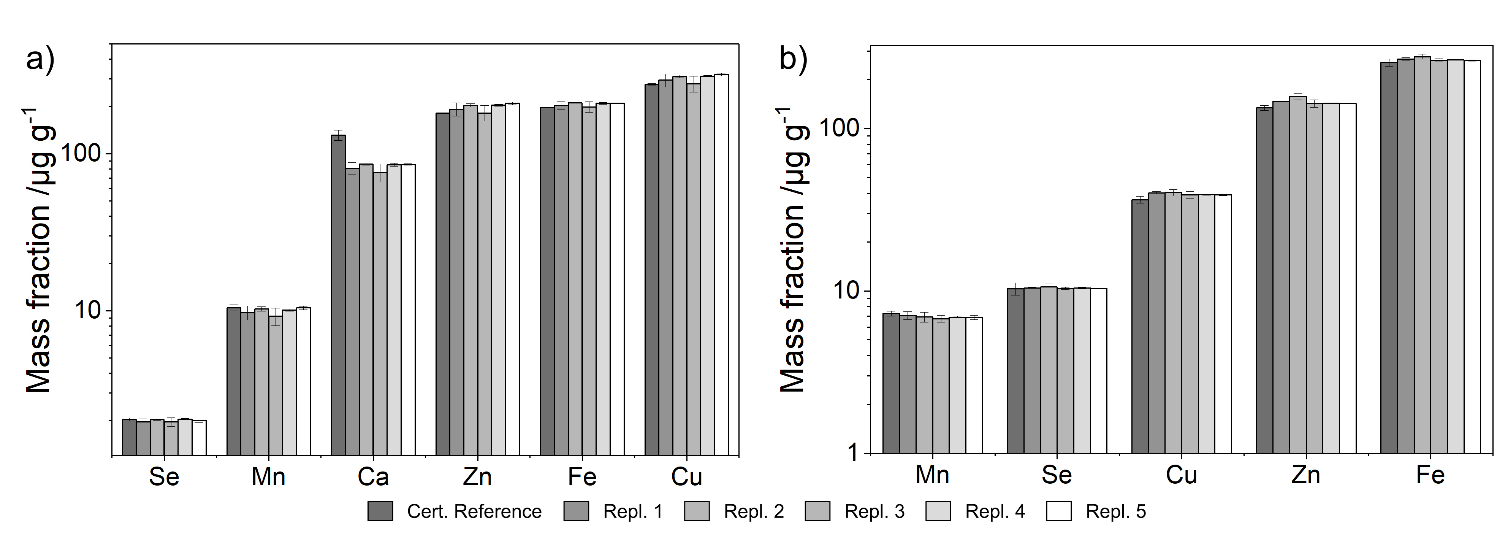


Pig kidney

Bovine liver

Figure S3: Mass fractions in different CRMs found by SA-TXRF using concentrated nitric acid as suspension reagent. a) NIST 1577c; b) ERM-BB186. (Data are mean values ± SD; n=3; measurement time 1,000 s; Sample weight: 20 mg; Suspension volume: 1mL).

## Overview


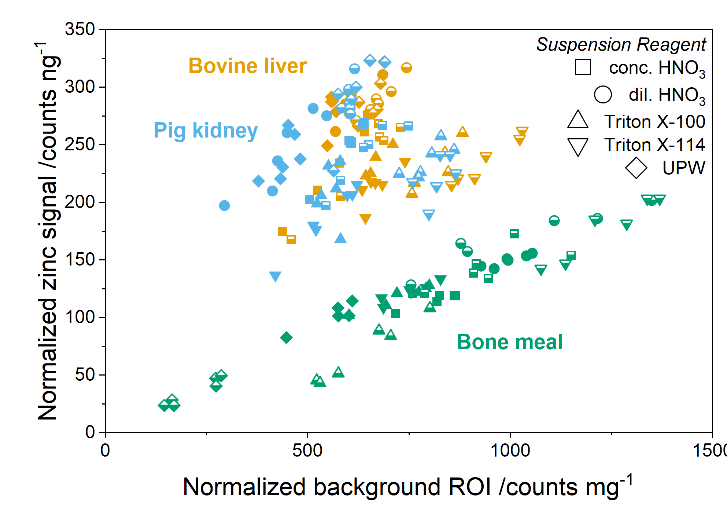


Figure S4: Comparison of normalized zinc signals (Zn K_α_ @ 8.367 keV) in relation to the background in the region of interest (ROI) for the five different suspension reagents and two tested sample amounts. The Zn signals are normalized to the sample amount and the zinc mass fraction of the respective reference material. The background signals are normalized to the sample amount. Each data point corresponds to an individually prepared sample carrier. Measurement time: 1,000 s.

## Recovery


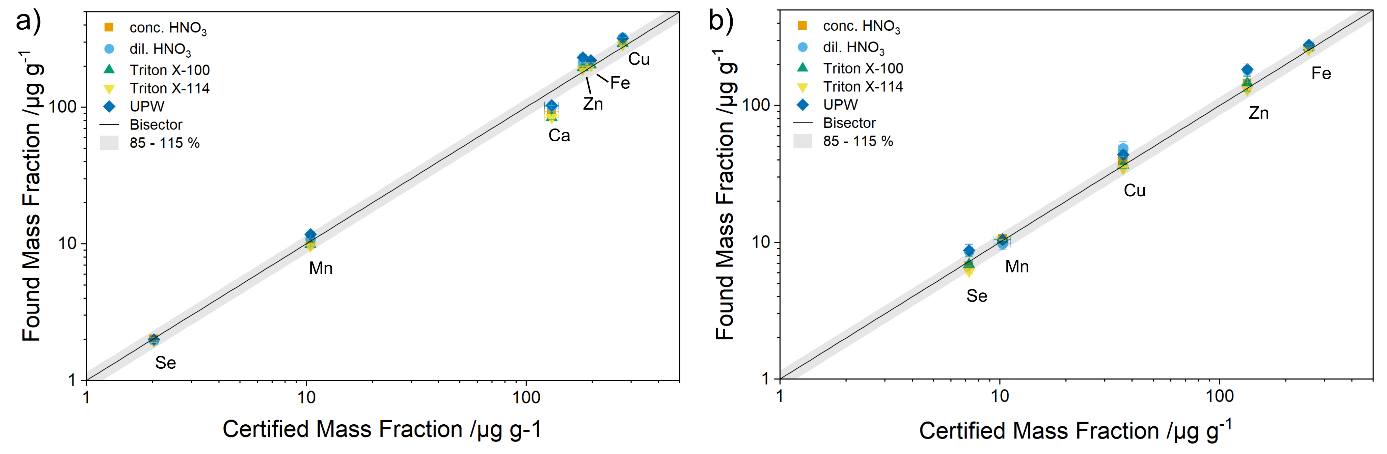


Figure S5: Recovery of mass fractions in different CRMs found by SA-TXRF using different suspension reagents. a) NIST 1577c; b) ERM-BB186. (Data are mean values ± expanded uncertainty with k=2; n=6; measurement time 1,000 s; Sample weight: 20 mg; Suspension volume: 1 mL)

## RSD

*
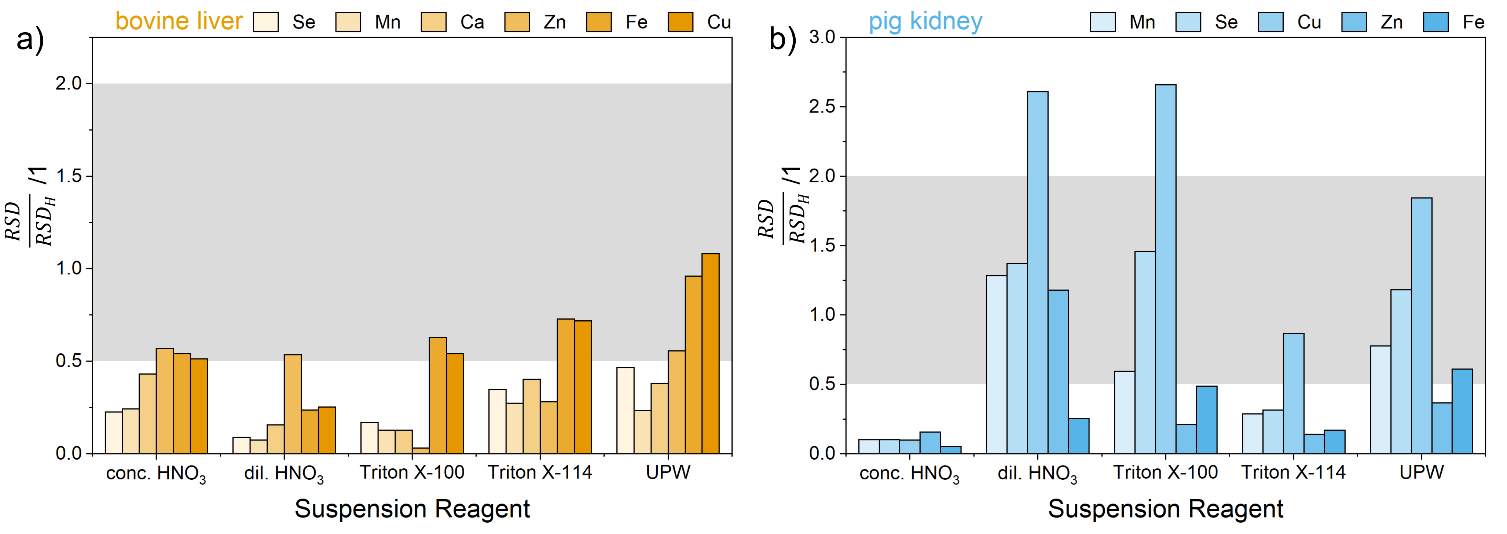
*

Figure S6: Ratio of observed RSD to RSD_H_ for a) NIST 1577c; b) ERM-BB186. (Sample weight: 20 mg; Suspension volume: 1 mL)

## SNR


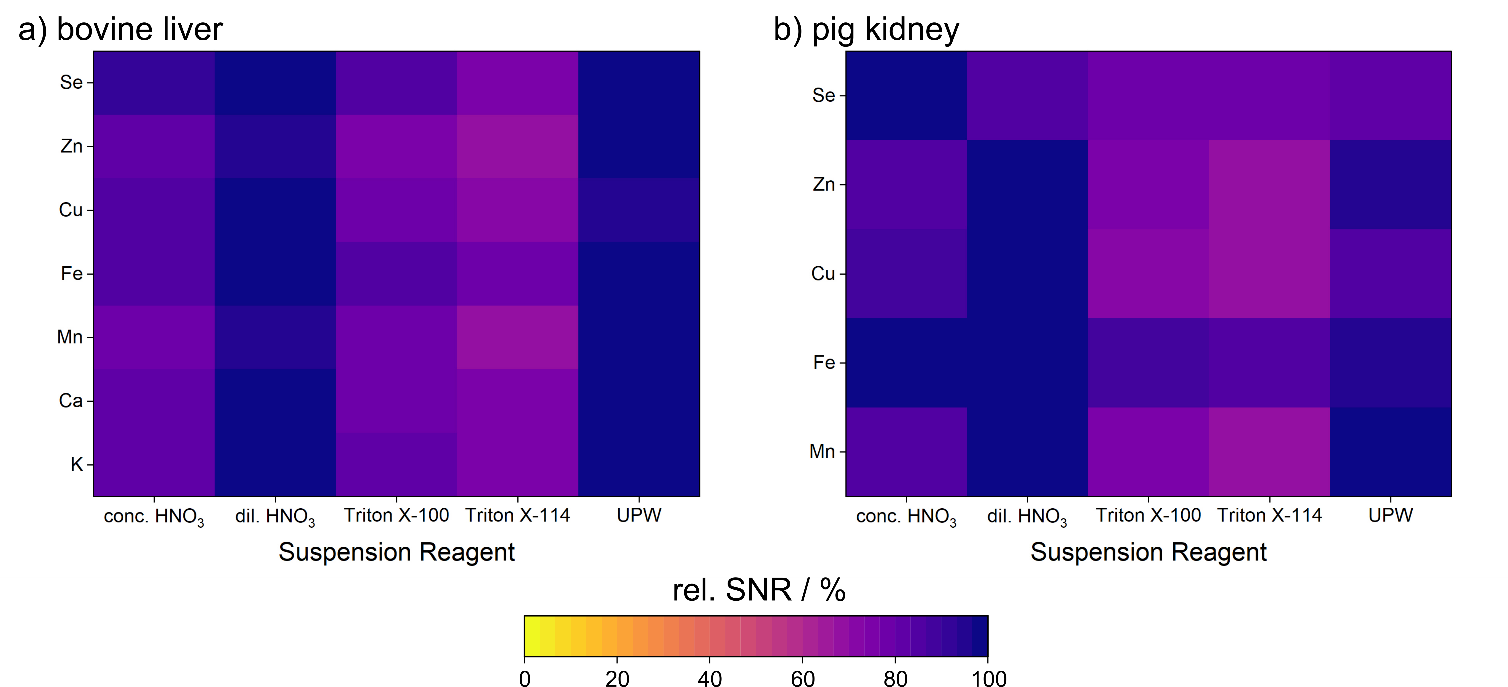


Figure S7: Relative signal-to-noise ratios (rel. SNRs) normalized to the maximum value observed for each element (set to 100%) for a) NIST 1577c; b) ERM-BB186; (Sample weight: 20 mg; Suspension volume: 1 mL).

## Limits of Quantification

Table S2: Minimum LOQs in µg L^-1^ found by SA-TXRF using different suspension reagents in NIST 1577c (n=6; measurement time 1.000 s; Sample weight: 10 and 20 mg; Suspension volume: 1 mL). Bold values indicate lowest LOQ found for each sample concentration.

| sample concentration | | | 10 mg mL^-1^ | | | | | 20 mg mL^-1^ | | | | |
| --- | --- | --- | --- | --- | --- | --- | --- | --- | --- | --- | --- | --- |
| Element | Line/ keV | Z | Conc. HNO_3_ | Dil. HNO_3_ | Triton X-100 | Triton X-114 | UPW | Conc. HNO_3_ | Dil. HNO_3_ | Triton X-100 | Triton X-114 | UPW |
| Ca | Kα@3.692 | 20 | 26 | **23** | 29 | 28 | 23 | 35 | **32** | 36 | 38 | 35 |
| Mn | Kα@5.900 | 25 | 7.7 | **6.3** | 8.7 | 8.6 | 6.4 | 9.6 | **8.9** | 10.5 | 10.9 | 9.8 |
| Fe | Kα@6.405 | 26 | 6.2 | **5.1** | 7.1 | 7.0 | 5.2 | 7.7 | **7.1** | 8.5 | 8.8 | 7.9 |
| Cu | Kα@8.046 | 29 | 4.1 | **3.3** | 4.6 | 4.6 | 3.5 | 5.0 | **4.7** | 5.6 | 5.9 | 5.2 |
| Zn | Kα@8.637 | 30 | 3.5 | **2.9** | 4.0 | 4.0 | 3.0 | 4.4 | **4.1** | 4.9 | 5.1 | 4.5 |
| Se | Kα@11.22 | 34 | 2.2 | **1.8** | 2.7 | 2.6 | 1.8 | 2.8 | **2.5** | 3.2 | 3.4 | 2.9 |

Table S3: Minimum LOQs in µg L^-1^ found by SA-TXRF using different suspension reagents in ERM-BB186 (n=6; measurement time 1.000 s; Sample weight: 10 and 20 mg; Suspension volume: 1 mL). Bold values indicate lowest LOQ found for each sample concentration.

| sample concentration | | | 10 mg mL^-1^ | | | | | 20 mg mL^-1^ | | | | |
| --- | --- | --- | --- | --- | --- | --- | --- | --- | --- | --- | --- | --- |
| Element | Line/ keV | Z | Conc. HNO_3_ | Dil. HNO_3_ | Triton X-100 | Triton X-114 | UPW | Conc. HNO_3_ | Dil. HNO_3_ | Triton X-100 | Triton X-114 | UPW |
| Mn | K_α_@5.900 | 25 | 7.6 | 6.3 | 7.7 | 8.2 | **6.0** | 9.7 | **9.7** | 10.5 | 10.9 | 10.1 |
| Fe | K_α_@6.405 | 26 | 6.2 | 5.0 | 6.2 | 6.5 | **4.8** | 7.9 | **7.7** | 8.5 | 8.7 | 8.1 |
| Cu | K_α_@8.046 | 29 | 3.7 | 3.0 | 3.8 | 4.1 | **3.0** | 4.8 | **4.7** | 5.3 | 5.5 | 5.1 |
| Zn | K_α_@8.637 | 30 | 3.3 | 2.7 | 3.4 | 3.6 | **2.6** | 4.3 | **4.1** | 4.7 | 4.8 | 4.5 |
| Se | K_α_@11.22 | 34 | 2.2 | 1.9 | 2.4 | 2.6 | **1.8** | 2.9 | **2.9** | 3.4 | 3.5 | 3.2 |

Table S4: Minimum LOQs in µg L^-1^ found by SA-TXRF using different suspension reagents in NIST 1486 (n=6; measurement time 1.000 s; Sample weight: 10 and 20 mg; Suspension volume: 1 mL). Bold values indicate lowest LOQ found for each sample concentration.

| sample concentration | | | 10 mg mL^-1^ | | | | | 20 mg mL^-1^ | | | | |
| --- | --- | --- | --- | --- | --- | --- | --- | --- | --- | --- | --- | --- |
| Element | Line/ keV | Z | Conc. HNO_3_ | Dil. HNO_3_ | Triton X-100 | Triton X-114 | UPW | Conc. HNO_3_ | Dil. HNO_3_ | Triton X-100 | Triton X-114 | UPW |
| K | K_α_@  3.314 | 19 | 256 | 227 | 166 | 173 | **150** | 392 | 367 | 248 | 272 | **245** |
| Fe | K_α_@  6.405 | 26 | 13.8 | 11.7 | 9.7 | 10.0 | **7.6** | 22.2 | 20.7 | 13.6 | 14.7 | **12.8** |
| Zn | K_α_@  8.637 | 30 | 6.4 | 5.7 | 4.9 | 5.0 | **3.5** | 10.6 | 9.9 | 6.7 | 7.0 | **5.9** |
| Sr | K_α_@  14.165 | 38 | 7.2 | 6.6 | 6.4 | 6.3 | **3.9** | 13.3 | 11.7 | 7.9 | 8.6 | **6.5** |
| Pb | L_α_@  10.551 | 82 | 5.5 | 4.4 | 4.4 | 4.2 | **3.1** | 9.1 | 5.8 | 5.3 | 5.8 | **4.8** |

## Greenness calculation

Individual criteria weights are set as default. The production of the internal standard is not taken into account here. Preparation of TXRF sample carriers is included in sample throughput.

Table S5: Input data and parameters used for calculation of AGREEprep scores by AGREEprep calculator (Version 0.91) for NIST 1577c and ERM-BB186.

| Criterion | Weight | Conc.  HNO_3_ | Dil.  HNO_3_ | Triton  X-100 | Triton  X-114 | UPW | EPA  3052 |
| --- | --- | --- | --- | --- | --- | --- | --- |
| 1 | 1 | Ex situ | | | | | |
| 2 | 5 | 1 mL | 0.14 mL | 0.01 mL | 0.01 mL | 0 mL | 10 mL |
| 3 | 2 | < 25 % | | | | | |
| 4 | 4 | 3.3 g | | | | | 27 g |
| 5 | 2 | 0.01 g | | | | | 0.25 g |
| 6 | 3 | 8 | | | | | 5 |
| 7 | 2 | 3 steps; semiautomated | | | | | 2 steps. auto |
| 8 | 4 | 15 Wh | | | | | 35 Wh |
| 9 | 2 | Spectrophotometry | | | | | |
| 10 | 3 | 3 | 3 | 3 | 3 | 0 | 4 |


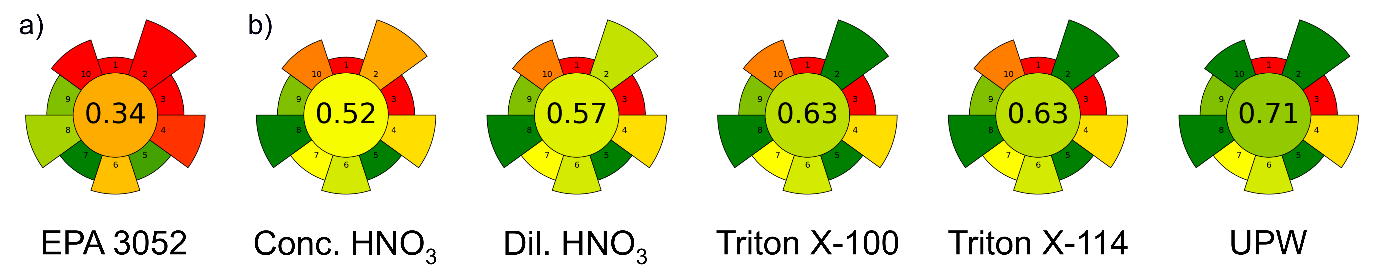


Figure S8: Calculated AGREEprep scores and pictograms for a) microwave-assisted sample digestion adapted from EPA 3052; and b) suspension-assisted sample preparation of bovine liver and pig kidney using different reagents. (Sample weight: 10 mg. sample volume: 1 mL; Further details used for calculation can be found in the SI.)

Table S6: Input data and parameters used for calculation of AGREEprep scores by AGREEprep calculator (Version 0.91) for NIST 1486.

| Criterion | Weight | Conc. HNO_3_ | Dil.  HNO_3_ | Triton  X-100 | Triton  X-114 | UPW | EPA  3052 |
| --- | --- | --- | --- | --- | --- | --- | --- |
| 1 | 1 | Ex situ | | | | | |
| 2 | 5 | 1 mL | 0.14 mL | 0.01 mL | 0.01 mL | 0 mL | 10 mL |
| 3 | 2 | < 25 % | | | | | |
| 4 | 4 | 3.3 g | | | | | 27 g |
| 5 | 2 | 0.01 g | | | | | 0.25 g |
| 6 | 3 | 12 | | | | | 5 |
| 7 | 2 | 2 steps; semiautomated | | | | | 2 steps. auto |
| 8 | 4 | 8.7 Wh | | | | | 35 Wh |
| 9 | 2 | Spectrophotometry | | | | | |
| 10 | 3 | 3 | 3 | 3 | 3 | 0 | 4 |

## Photos


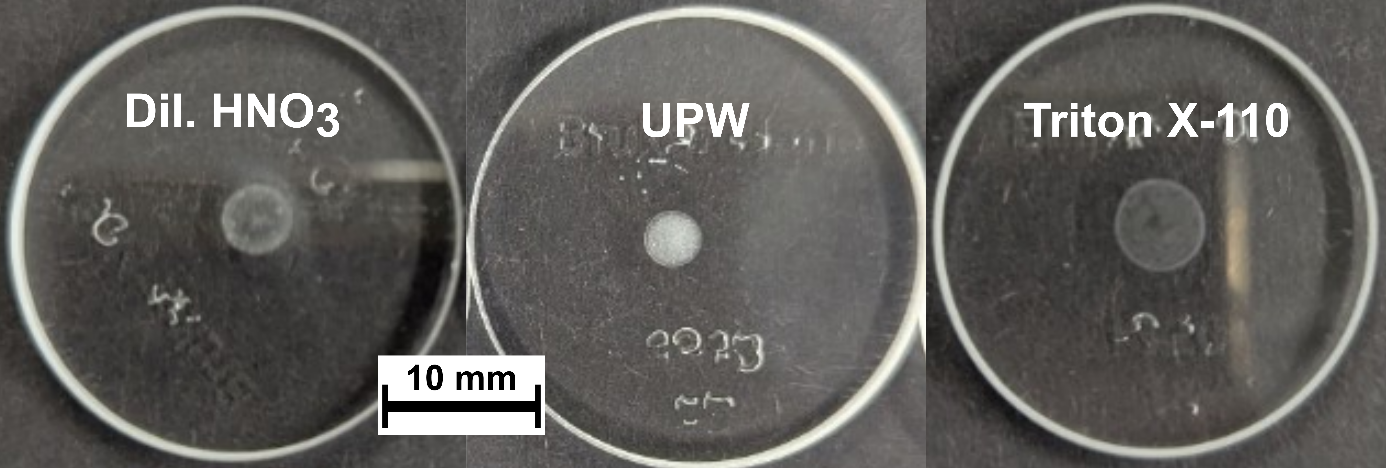


Figure S9: Prepared sample carriers using different suspension reagents in NIST 1486 (Sample weight: 10 mg; Suspension volume: 1 mL).

**References**

1. Bolin FP, Preuss LE, Taylor RC, Ference RJ (1989) Refractive index of some mammalian tissues using a fiber optic cladding method. Appl Opt, AO 28:2297–2303. https://doi.org/10.1364/AO.28.002297
